# Interventions to Reduce Antibiotic Prescribing in Small Animal Veterinary Practice: Evidence and Clinical Impact

**DOI:** 10.3390/antibiotics15090916

**Published:** 2026-09-17

**Authors:** Ionela Popa, Ionica Iancu, Alexandru Gligor, Kalman Imre, Emil Tîrziu, Timea Bochiș, Călin Pop, Janos Degi, Andrei Alexandru Ivan, Michael Dahma, Ana-Maria Plotuna, Sebastian Alexandru Popa, Marius Pentea, Viorel Herman, Ileana Nichita

**Affiliations:** 1 Department of Semiology, Faculty of Veterinary Medicine, University of Life Sciences “King Mihai I” from Timisoara, 300645 Timisoara, Romania; ionela.popa@usvt.ro (I.P.); timea.bochis@usvt.ro (T.B.); calinpop@usvt.ro (C.P.); 2 Doctoral School “Veterinary Medicine”, University of Life Sciences “King Mihai I” from Timisoara, 300645 Timisoara, Romania; andreialexandru.ivan.fmv@usvt.ro (A.A.I.); michael.dahma@usvt.ro (M.D.); anamaria.plotuna@usvt.ro (A.-M.P.); 3 Department of Infectious Diseases and Preventive Medicine, Faculty of Veterinary Medicine, University of Life Sciences “King Mihai I” from Timisoara, 300645 Timisoara, Romania; janosdegi@usvt.ro (J.D.); viorelherman@usvt.ro (V.H.); 4 Department of Animal Production and Veterinary Public Health, University of Life Sciences “King Mihai I” from Timisoara, 300645 Timisoara, Romania; alexandru.gligor@usvt.ro (A.G.); kalmanimre@usvt.ro (K.I.); emiltirziu@usvt.ro (E.T.); sebastian.popa@usvt.ro (S.A.P.); ileananichita@usvt.ro (I.N.); 5 Department of Anatomy, University of Life Sciences “King Mihai I” from Timisoara, 300645 Timisoara, Romania; mariuspentea@usvt.ro; 6 Academy of Romanian Scientists, Str. Ilfov Nr. 3, Sector 5, 050044 Bucharest, Romania

**Keywords:** antimicrobial stewardship, companion animals, antimicrobial resistance, diagnostic stewardship, One Health

## Abstract

Antimicrobial resistance represents a major One Health challenge affecting human, animal, and environmental health, and responsible antimicrobial use in companion animal medicine has become increasingly important. This narrative review synthesizes current evidence on antimicrobial stewardship interventions aimed at improving antibiotic prescribing in small animal veterinary practice. The review discusses the clinical, behavioral, owner-related, organizational, and regulatory factors that influence prescribing decisions, with emphasis on diagnostic uncertainty, access to microbiological testing, communication with pet owners, and practice-level prescribing culture. Key stewardship strategies include evidence-based clinical guidelines, diagnostic stewardship, education and training, audit and feedback, electronic clinical decision-support systems, antimicrobial resistance surveillance, infection prevention, and multifaceted implementation programs. Evidence from predominantly observational and before-and-after studies suggests that these interventions may be associated with reductions in unnecessary antimicrobial prescribing and improvements in prescribing appropriateness, although the strength of causal evidence remains limited. However, further research is needed because current studies differ in methodology, often have short follow-up periods, include limited data from routine primary veterinary practices, and rarely demonstrate a direct reduction in antimicrobial resistance. Future progress will depend on standardized outcome measures, integration of digital health technologies, rapid diagnostics, artificial intelligence, antimicrobial resistance surveillance, and stronger One Health collaboration. Overall, antimicrobial stewardship in companion animal medicine should be regarded as a continuous quality-improvement process designed to preserve antimicrobial efficacy while maintaining optimal patient care.

## 1. Introduction

Antimicrobial resistance (AMR) is recognized as one of the most significant global threats to human and animal health [1,2,3,4,5,6,7,8]. The increasing prevalence of multidrug-resistant bacterial pathogens has prompted international organizations, including the World Health Organization (WHO), the World Organisation for Animal Health (WOAH), and the Food and Agriculture Organization (FAO), to recognize antimicrobial stewardship as a cornerstone of the global One Health strategy aimed at preserving antimicrobial efficacy across human, animal, and environmental health sectors [1,2]. The One Health concept emphasizes that human health, animal health, and environmental health are interconnected and that antimicrobial resistance can emerge, persist, and spread across these interconnected systems [3,4,9,10,11,12,13]. Resistant microorganisms and resistance genes may circulate between humans, animals, food systems, and environmental reservoirs through multiple pathways, including direct contact, occupational exposure, contamination of shared environments, and movement of microbial populations. Therefore, antimicrobial stewardship represents a coordinated global responsibility requiring collaboration between veterinary medicine, human healthcare, public health authorities, environmental scientists, and policymakers [10].

The increasing global burden of antimicrobial resistance has stimulated the development of international action plans aimed at preserving antimicrobial efficacy and reducing inappropriate antimicrobial exposure. These initiatives recognize responsible antimicrobial use as one of the most effective strategies for slowing resistance development, while maintaining the ability to treat bacterial infections effectively in both human and veterinary medicine. Within this framework, companion animal medicine has received increasing attention because pets live in close proximity to humans and may contribute to the exchange of antimicrobial-resistant organisms and resistance determinants within households and communities [14,15,16].

Although companion animals account for a smaller proportion of antimicrobial consumption than food-producing animals, antimicrobial use and resistance in both sectors represent important One Health concerns, although the surveillance systems, prescribing contexts, and stewardship approaches differ substantially. In food-producing animals, antimicrobial consumption has been monitored through European population-level surveillance systems, and substantial reductions in antimicrobial sales have been documented. Among 25 European countries with continuous data, overall sales of veterinary antibiotics decreased by 53.0% between 2011 and 2022. During the same period, sales of fluoroquinolones, third- and fourth-generation cephalosporins, other quinolones, and polymyxins decreased by 24.7%, 49.0%, 89.7%, and 81.0%, respectively [17]. These reductions have occurred alongside regulatory measures, surveillance, prudent-use policies, improved disease prevention, biosecurity, and other interventions aimed at reducing unnecessary antimicrobial exposure in food-producing animals.

In companion animals, antimicrobial-use and resistance surveillance remains less standardized at the European level, while prescribing decisions are primarily made at the individual-patient level. Nevertheless, available evidence demonstrates substantial antimicrobial exposure and clinically relevant resistance in dogs and cats. In a cross-sectional study conducted in three European countries, 19% of sampled companion animals had received at least one antimicrobial treatment during the preceding six months. Amoxicillin–clavulanate accounted for 27% of antimicrobial treatments, while broad-spectrum antimicrobials and critically important antimicrobials represented 83% and 71% of treatments, respectively. Among 282 *Escherichia coli* isolates obtained from these animals, 27% were resistant to at least one antimicrobial, with resistance rates of 18% for ampicillin, 15% for sulfamethoxazole, and 14% for tetracycline; 13% of isolates were multidrug-resistant [18].

Evidence from previous studies conducted in western Romania further illustrates the occurrence of antimicrobial resistance among companion-animal pathogens. *Staphylococcus pseudintermedius* was identified in 40% of dogs with otitis externa and 21.1% of healthy dogs, with tetracycline resistance rates of 37.5% and 25%, respectively, while methicillin-resistant *S. pseudintermedius* (MRSP) was detected in 1.2% of dogs with otitis [19]. Another study from the same region found penicillin resistance in 65% of *Staphylococcus aureus* isolates from dogs with otitis and 54.3% of isolates from healthy dogs, while multidrug resistance was observed in 25% and 16% of isolates, respectively [20]. These findings further support the presence of clinically relevant antimicrobial resistance among companion animals, including apparently healthy dogs.

For comparison, recent European surveillance data for indicator *E. coli* from pigs reported resistance rates of 35.6% for ampicillin, 34.8% for sulfamethoxazole, and 41.5% for tetracycline [21]. However, the comparatively lower antimicrobial consumption and resistance levels observed in companion animals should not be interpreted as indicating a negligible AMR risk, given the close and sustained contact between pets and humans and the potential for exchange of resistant bacteria and resistance determinants.

Close physical contact between pets and humans also creates opportunities for bidirectional transmission of antimicrobial-resistant bacteria and resistance genes, emphasizing the public health relevance of prescribing practices in companion animal medicine [14,15,16]. Companion animals may act as reservoirs for clinically important antimicrobial-resistant bacteria, including MRSP, methicillin-resistant *Staphylococcus aureus* (MRSA), extended-spectrum β-lactamase (ESBL)-producing *Escherichia coli*, multidrug-resistant *Enterococcus* species, and other resistant opportunistic pathogens. Increasing evidence suggests that bacterial strains and resistance genes can be shared between companion animals and their owners, although the precise contribution of pets to the overall human AMR burden remains difficult to quantify. Nevertheless, these findings highlight the importance of optimizing antimicrobial prescribing practices in veterinary medicine as part of broader One Health resistance-control strategies [7,19,20,22,23,24,25,26].

Veterinary antimicrobial stewardship (AMS) aims to optimize antibiotic use by ensuring that antimicrobial therapy is necessary, evidence-based, and tailored to the individual patient. Beyond reducing antimicrobial consumption, stewardship seeks to maximize clinical efficacy while minimizing adverse drug reactions, selection pressure for resistant microorganisms, disruption of the normal microbiota, and unnecessary healthcare costs [27]. Importantly, antimicrobial stewardship should not be interpreted solely as a strategy for reducing antimicrobial use. Instead, it represents a comprehensive approach focused on improving prescribing quality through selection of the most appropriate antimicrobial agent, correct dosing, appropriate route of administration, optimal treatment duration, and continuous reassessment of therapeutic necessity. These principles aim to balance effective infection management with minimization of unnecessary antimicrobial exposure [27,28].

A key objective of modern stewardship is the preservation of critically important antimicrobials that represent essential therapeutic options for severe or multidrug-resistant infections. Inappropriate use of broad-spectrum or highest-priority antimicrobial agents may accelerate the selection of resistant organisms and compromise future treatment options. Consequently, current stewardship frameworks emphasize targeted therapy, antimicrobial spectrum optimization, appropriate diagnostic investigation, and timely modification or discontinuation of treatment when clinical or microbiological evidence supports such decisions [28].

Successful antimicrobial stewardship requires integration of multiple complementary components, including evidence-based prescribing guidelines, diagnostic stewardship, infection prevention measures, antimicrobial resistance surveillance, continuing professional education, and effective communication with pet owners. Increasingly, implementation science and behavioral science approaches have been incorporated into stewardship programs because prescribing decisions are influenced not only by clinical evidence but also by cognitive biases, professional experience, social expectations, organizational culture, and healthcare system factors. Understanding these determinants is essential for developing interventions capable of producing sustained changes in antimicrobial prescribing behavior [29].

This review summarizes the current evidence on interventions designed to reduce antibiotic prescribing in small animal veterinary practice and evaluates their impact on prescribing behavior, clinical outcomes, and future stewardship strategies. In addition, the review critically discusses the strengths and limitations of currently available evidence and identifies emerging opportunities for improving antimicrobial stewardship implementation in companion animal practice. Particular emphasis is placed on interventions targeting different levels of antimicrobial prescribing, including educational strategies, clinical guidelines, diagnostic approaches, audit and feedback systems, electronic decision-support tools, and multifaceted stewardship programs. Furthermore, this review evaluates current knowledge gaps, methodological limitations, and future opportunities involving digital health technologies, artificial intelligence, advanced diagnostics, and international collaboration to strengthen antimicrobial stewardship within companion animal medicine.

## 2. Factors Influencing Antibiotic Prescribing

Antibiotic prescribing decisions are influenced by multiple clinical and non-clinical factors. Diagnostic uncertainty, clinician experience, owner expectations, financial constraints, and limited access to microbiological testing often encourage empirical prescribing. Time pressure and concerns about disease progression may also contribute to unnecessary antimicrobial use [30,31,32].

The availability of diagnostic resources strongly influences the degree of clinical uncertainty encountered during routine consultations. Veterinary practices equipped with in-house cytology, microscopy, hematology, or rapid point-of-care diagnostic tests may be better able to differentiate bacterial from non-bacterial diseases during the initial evaluation, thereby reducing reliance on empirical antimicrobial therapy. Conversely, practices with limited diagnostic infrastructure often face greater uncertainty, particularly when referral laboratories are not readily accessible or when obtaining additional diagnostic information would substantially delay treatment decisions [33].

Owner-related factors also substantially influence prescribing behavior. Expectations for immediate treatment, concerns regarding animal welfare, previous positive experiences with antimicrobial therapy, financial limitations, and reluctance to pursue additional diagnostic investigations may all affect therapeutic decision-making. Effective communication between veterinarians and pet owners has therefore become an increasingly recognized component of antimicrobial stewardship, helping owners understand when antimicrobial therapy is unnecessary or when additional diagnostic testing is likely to improve treatment outcomes [34,35].

Effective antimicrobial stewardship increasingly recognizes communication skills as an important component of veterinary practice. Evidence from companion-animal owner perspectives indicates that clear communication, trust-building, and shared decision-making can facilitate discussions about antimicrobial use and increase openness to alternatives to antimicrobial treatment. Veterinarians who clearly explain diagnostic uncertainty, discuss the expected course of disease, outline the potential risks associated with unnecessary antimicrobial use, and actively involve pet owners in treatment decisions may therefore support more informed antimicrobial stewardship discussions. However, evidence directly demonstrating that structured communication training improves antimicrobial prescribing quality or client satisfaction in companion-animal practice remains limited. Further research is needed to determine whether targeted communication interventions can produce measurable improvements in prescribing outcomes while maintaining effective veterinarian–client relationships [36].

Socioeconomic considerations may further shape owner expectations and influence antimicrobial prescribing. Financial constraints may limit acceptance of microbiological culture, antimicrobial susceptibility testing, advanced imaging, or repeated follow-up consultations, potentially contributing to empirical treatment when diagnostic uncertainty remains. In addition, qualitative evidence suggests that clear veterinarian–client communication and shared decision-making may facilitate discussions about alternatives to antimicrobial treatment and support owner engagement in treatment decisions. However, the extent to which these communication approaches directly modify antimicrobial prescribing or acceptance of specific stewardship interventions remains insufficiently established in companion-animal practice [36].

Behavioral factors have increasingly been recognized as major determinants of prescribing decisions. Cognitive biases, including risk aversion, overestimation of treatment benefits, fear of missing bacterial infections, and defensive prescribing practices, may contribute to unnecessary antimicrobial administration despite the availability of clinical guidelines. Understanding these behavioral drivers is essential for designing effective stewardship interventions [37,38].

Evidence concerning behavioural and organisational determinants of antimicrobial prescribing is substantially stronger in human healthcare than in companion animal medicine. Therefore, some of the mechanisms discussed below are derived from human healthcare research and should be interpreted as potentially transferable rather than directly demonstrated in veterinary practice. Companion animal prescribing occurs within a distinct veterinarian–animal–owner relationship, in which clinical decision-making is additionally influenced by owner expectations, willingness and ability to pay for diagnostics or treatment, adherence to treatment recommendations, and the veterinarian–client relationship.

Accordingly, throughout this review, evidence from human healthcare is used primarily to provide contextual or conceptual support where veterinary evidence is limited, rather than to imply that equivalent effects have been directly demonstrated in companion animal practice. Where veterinary-specific evidence is available, it is preferentially used to support statements concerning antimicrobial prescribing and stewardship in veterinary medicine.

In companion animal practice, these behavioural and organisational determinants may operate differently from those described in human healthcare. Available veterinary evidence indicates that prescribing decisions can be influenced by owner expectations, treatment costs, diagnostic availability, professional interactions, clinic culture, and veterinarians’ perceptions of antimicrobial risks. Consequently, human-derived behavioural frameworks should be regarded as complementary to, rather than a substitute for, evidence generated in companion animal practice [35,36,39].

Organizational factors also play an important role. Practice policies, availability of diagnostic equipment, consultation duration, workload, access to continuing professional education, and local antimicrobial stewardship culture may significantly influence prescribing behavior. Consequently, stewardship interventions targeting organizational systems, rather than individual clinicians alone, are increasingly recognized as necessary for achieving sustained improvements in antimicrobial prescribing quality [40].

Practice-specific antimicrobial formularies and locally adapted prescribing guidelines may additionally standardize therapeutic decision-making by incorporating regional antimicrobial resistance patterns, local pathogen prevalence, and drug availability. Regular revision of these recommendations is essential because bacterial epidemiology and antimicrobial susceptibility profiles evolve over time, requiring stewardship programs to remain responsive to changing clinical circumstances [41].

In addition to clinician, owner, and organization-related determinants, antimicrobial prescribing is increasingly influenced by external regulatory and professional factors. National antimicrobial stewardship policies, restrictions on the use of highest-priority critically important antimicrobials, professional guidelines, and mandatory prescribing documentation may all contribute to more standardized prescribing practices. Antimicrobial-use surveillance and reporting systems can provide important information for benchmarking prescribing practices and monitoring changes in antimicrobial use. At the European level, regulatory requirements have established frameworks for the collection, reporting and interpretation of antimicrobial sales and use data in animals [42,43,44,45,46].

Patient-related and clinical characteristics may influence antimicrobial treatment decisions, particularly in complex or critically ill patients. In veterinary practice, antimicrobial selection should take into account the species and clinical context, the suspected site and severity of infection, diagnostic findings, previous antimicrobial exposure and the risk of antimicrobial resistance [47,48,49].

Finally, prescribing decisions should be viewed as dynamic processes that evolve throughout patient management. Initial empirical therapy should be reassessed as additional clinical information, laboratory results, or microbiological findings become available, allowing discontinuation, de-escalation, or modification of antimicrobial therapy whenever appropriate. Regular reassessment represents a fundamental stewardship principle that minimizes unnecessary antimicrobial exposure while maintaining optimal patient care (Table 1) [48].

To translate these considerations into practice, antimicrobial stewardship should combine diagnostic support, evidence-based prescribing guidance, clinical decision-support tools, and regular audit and feedback to promote appropriate antimicrobial selection and timely treatment reassessment [50,51,52,53,54]. These measures should be complemented by clear veterinarian–owner communication and preventive strategies aimed at reducing the occurrence of infections and the subsequent need for antimicrobial treatment. Importantly, stewardship interventions should be adapted to the resources and workflow of individual practices and integrated into routine clinical decision-making rather than relying solely on individual prescriber awareness.

**Table 1:** Major factors influencing antimicrobial prescribing decisions in companion animal practice.

Factor Category	Main Determinants	Potential Impact on Antimicrobial Prescribing	Stewardship Opportunities
Clinical factors	Diagnostic uncertainty, disease severity, lack of microbiological confirmation, previous treatment failure [33,47,55]	Increased empirical antimicrobial prescribing	Diagnostic stewardship, rapid diagnostics, treatment reassessment [56,57,58,59,60] *
Veterinarian-related factors	Clinical experience, prescribing habits, risk perception, cognitive biases, defensive prescribing [30,34,35,38,40] *	Variation in prescribing decisions	Education, audit and feedback, decision-support systems [50] *, [51,52,61,62,63,64,65,66] *
Owner-related factors	Expectations for antibiotics, concerns regarding disease progression, financial limitations [35,36]	Pressure toward unnecessary treatment	Improved veterinarian–owner communication, shared decision-making [35,36]
Organizational factors	Practice culture, workload, consultation time, access to diagnostics [34,35,37,40] *	Differences between clinics and institutions	Institutional policies, stewardship champions, workflow optimization [39,51,67]
Regulatory factors	Antimicrobial availability, national regulations, prescribing restrictions [42,43,44,45,46]	Differences between countries	Harmonized guidelines and surveillance systems [43,44,45,46,49,68]

* Evidence marked with an asterisk is derived from human healthcare literature. These studies are included where direct evidence from companion-animal practice is limited and are used to inform transferable behavioral, organizational, diagnostic, or antimicrobial stewardship concepts; they should not be interpreted as direct evidence for companion-animal practice.

## 3. Antimicrobial Stewardship in Companion Animal Medicine

Antimicrobial stewardship encompasses coordinated interventions that promote responsible antimicrobial use while preserving treatment efficacy and minimizing resistance development. International organizations such as the International Society for Companion Animal Infectious Diseases (ISCAID), the World Small Animal Veterinary Association (WSAVA), the British Small Animal Veterinary Association (BSAVA), the Federation of European Companion Animal Veterinary Associations (FECAVA), and WOAH have developed evidence-based recommendations to guide antimicrobial prescribing in companion animal medicine [33,49,67].

More recently, the European Network for Optimization of Veterinary Antimicrobial Therapy (ENOVAT) has developed evidence-based guidelines for antimicrobial use in companion animals, including recommendations for antimicrobial use in canine acute diarrhoea and surgical antimicrobial prophylaxis in dogs and cats [53,54]. National guidance also contributes to antimicrobial stewardship. For example, Sweden has developed specific recommendations for antimicrobial use in dogs through the Swedish Medical Products Agency, complemented by guidance from the Swedish Veterinary Association [69].

These international recommendations reflect a growing recognition that antimicrobial stewardship in veterinary medicine must extend beyond individual prescribing decisions and become integrated into broader healthcare quality-improvement systems. Unlike approaches focused exclusively on antimicrobial reduction, contemporary stewardship frameworks emphasize optimization of antimicrobial use by balancing therapeutic efficacy, patient safety, resistance mitigation, and responsible resource utilization. This paradigm shift is particularly important in companion animal medicine, where clinical decisions occur within the complex context of individual patient care, owner expectations, and the wider One Health implications of antimicrobial resistance [49].

These recommendations emphasize that antimicrobial prescribing should be based on accurate diagnosis, assessment of infection severity, likely bacterial pathogens, expected antimicrobial susceptibility, pharmacokinetic and pharmacodynamic principles, and careful evaluation of the potential risks and benefits of treatment. Whenever possible, antimicrobial therapy should be supported by microbiological culture and antimicrobial susceptibility testing, particularly in recurrent, complicated, or non-responsive infections. Such an evidence-based approach reduces unnecessary empirical prescribing while improving treatment precision [49].

An additional principle of modern antimicrobial stewardship is the integration of pharmacological optimization into prescribing decisions. Appropriate antimicrobial selection requires consideration not only of bacterial susceptibility but also of drug penetration into the infected tissue, pharmacokinetic variability between species, patient-specific factors affecting drug disposition, and pharmacodynamic targets associated with bacterial eradication. In companion animal practice, where evidence-based dosing information remains incomplete for some antimicrobial agents and clinical conditions, continued research into veterinary pharmacology represents an important component of stewardship development [70].

Modern antimicrobial stewardship programs extend beyond the publication of clinical guidelines and instead incorporate continuous quality-improvement strategies that seek to optimize prescribing behavior over time. Stewardship is therefore increasingly viewed as an ongoing institutional process involving regular evaluation of prescribing practices, implementation of targeted interventions, monitoring of outcomes, and repeated reassessment of program effectiveness [70].

Successful antimicrobial stewardship requires a multidisciplinary approach involving veterinarians, microbiologists, clinical pharmacologists, veterinary nurses, diagnostic laboratories, hospital administrators, and pet owners. The effectiveness of stewardship programs depends not only on guideline availability but also on organizational support, continuous education, surveillance systems, and regular evaluation of prescribing practices [70].

The contribution of veterinary nurses and support staff is particularly important because they frequently represent the primary point of contact for monitoring treatment response, reinforcing administration instructions, and communicating with owners throughout the treatment process. Their involvement may improve adherence to prescribed antimicrobial regimens and facilitate early recognition of adverse effects or clinical deterioration requiring reassessment of therapy. Incorporating veterinary nurses into stewardship teams may therefore enhance the practical implementation of stewardship principles within routine clinical workflows [71,72].

Diagnostic laboratories also play an increasingly important role beyond providing individual culture and susceptibility results. By aggregating microbiological data across cases, laboratories can contribute to regional antimicrobial resistance surveillance, identify emerging resistance trends, and support the development of locally relevant prescribing recommendations. Improved communication between veterinary practices and diagnostic laboratories may further enhance the clinical interpretation of laboratory findings and promote more targeted antimicrobial use [68,73,74].

Surveillance systems represent another essential component of antimicrobial stewardship. Continuous monitoring of antimicrobial consumption, prescribing patterns, and antimicrobial resistance enables veterinary institutions to identify emerging trends, evaluate stewardship interventions, and benchmark prescribing performance over time. Standardized surveillance data also facilitate comparisons between practices, regions, and countries, thereby supporting national and international antimicrobial resistance control strategies [73,75].

Effective surveillance requires standardized definitions, consistent data collection methods, and meaningful indicators that allow comparison across different veterinary settings. Unlike human healthcare, where national antimicrobial consumption and resistance monitoring systems are relatively established, veterinary surveillance remains heterogeneous in many regions. Expansion of coordinated veterinary surveillance networks represents an important priority for improving understanding of antimicrobial use patterns and evaluating the population-level impact of stewardship interventions [73,75].

Regulatory measures have become an increasingly important component of antimicrobial stewardship in Europe. Regulation (EU) 2019/6 on veterinary medicinal products established a strengthened legal framework for the prudent use of antimicrobials. In particular, Article 107 prohibits routine antimicrobial use and the use of antimicrobials to compensate for poor hygiene or inadequate animal husbandry, restricts prophylactic use to exceptional circumstances, and limits metaphylactic use to situations in which the risk of disease spread is high and no appropriate alternatives are available. The Regulation also provides a legal framework for restricting the use of specific antimicrobials and for reserving certain antimicrobials for the treatment of specific human infections [42].

Regulation (EU) 2019/6 has also strengthened European antimicrobial-use surveillance by requiring Member States to collect and report data on the sales and use of antimicrobial medicinal products in animals. These requirements were further specified through Commission Delegated Regulation (EU) 2021/578 and Commission Implementing Regulation (EU) 2022/209, which established requirements and standardized formats for the collection and reporting of antimicrobial-use data to the European Medicines Agency (EMA) [43,44]. The European Medicines Agency subsequently developed standardized denominators and indicators to facilitate comparable assessment of antimicrobial sales and use across countries and over time [45].

The transition from voluntary European Surveillance of Veterinary Antimicrobial Consumption (ESVAC) monitoring to mandatory reporting under the Veterinary Medicinal Products Regulation represents an important development for antimicrobial stewardship. Since 2024, EU and EEA countries have reported antimicrobial sales and use data to the EMA through the Antimicrobial Sales and Use Platform, with annual European Sales and Use of Antimicrobials for Veterinary Medicine (ESUAvet) reports providing increasingly standardized information on antimicrobial consumption [46]. These data can support stewardship by identifying temporal trends, differences between countries, and potential areas for targeted intervention and benchmarking. However, species-level reporting is being implemented progressively, and mandatory reporting of antimicrobial use in dogs and cats is scheduled to begin in 2030. Therefore, development of robust, species-specific antimicrobial-use surveillance and benchmarking systems for companion animals remains an important research and policy priority [43,46].

Despite increasing awareness and strengthening regulatory frameworks, implementation of AMS remains inconsistent because of resource limitations, variability in clinical settings, differences in local prescribing cultures, and heterogeneity in antimicrobial-use surveillance systems [46,73,75].

Furthermore, implementation barriers differ considerably between referral hospitals and primary care practices, indicating that stewardship strategies should be adapted to local clinical contexts rather than relying on a universal implementation model [73,75].

The successful translation of stewardship recommendations into clinical practice requires recognition that implementation barriers are context dependent. Interventions developed in academic referral centers may not be directly transferable to first-opinion practices, where differences in staffing, financial resources, consultation duration, diagnostic availability, and patient populations may substantially influence feasibility. Implementation strategies should therefore be designed using principles from implementation science, with assessment of local barriers, available resources, stakeholder engagement, and measurable outcomes before widespread adoption [73,75].

Resource limitations remain particularly challenging for smaller first-opinion practices, where financial constraints, limited access to microbiological diagnostics, workforce shortages, and restricted consultation times may reduce opportunities for comprehensive stewardship implementation. In contrast, referral hospitals often possess greater diagnostic capabilities but may encounter more complex clinical cases requiring individualized antimicrobial decision-making. Consequently, stewardship interventions should be sufficiently flexible to accommodate differences in patient populations, available infrastructure, and organizational capacity [76].

In smaller companion animal practices, feasible stewardship interventions may include simplified prescribing guidelines, delayed prescribing strategies, improved client communication tools, regular case discussions, and selective use of diagnostic testing in high-risk cases. Such approaches may achieve meaningful improvements without requiring extensive financial investment or complex digital infrastructure. Conversely, larger referral hospitals may benefit from more advanced interventions, including antimicrobial stewardship committees, electronic prescribing systems, prospective audit and feedback, and dedicated surveillance programs [76].

Successful implementation additionally depends on organizational leadership and institutional commitment. Practices that establish clear antimicrobial prescribing policies, designate stewardship champions, provide regular clinician feedback, and promote a culture of continuous quality improvement generally achieve greater and more sustainable improvements in prescribing behavior than institutions relying solely on passive dissemination of clinical guidelines. Leadership engagement has therefore emerged as one of the strongest predictors of successful stewardship implementation across both human and veterinary healthcare settings [76].

The presence of antimicrobial stewardship champions may be particularly valuable because these individuals can facilitate communication between clinicians, promote guideline adoption, coordinate educational activities, and encourage engagement with prescribing audits. Importantly, stewardship leadership should be perceived as supportive rather than punitive, as excessive focus on restriction or surveillance alone may generate resistance among clinicians and reduce long-term participation [76].

Increasingly, antimicrobial stewardship is also being integrated with broader infection prevention and biosecurity programs. Effective hand hygiene, environmental cleaning, isolation of infectious patients, vaccination strategies, surgical asepsis, and hospital infection-control measures all reduce the incidence of bacterial infections and consequently decrease the need for antimicrobial therapy. This integrated approach recognizes that preventing infections represents one of the most effective methods for reducing antimicrobial consumption while simultaneously limiting opportunities for the emergence and spread of antimicrobial-resistant organisms [73,77,78].

The integration of infection prevention and control (IPC) measures with antimicrobial stewardship represents a fundamental component of the One Health approach. Preventing infections reduces the need for antimicrobial exposure at both individual and population levels, thereby decreasing selective pressure for resistance development. In companion animal hospitals, implementation of standardized IPC protocols—including patient isolation procedures, environmental hygiene strategies, appropriate handling of contaminated materials, and surveillance of healthcare-associated infections—can substantially complement antimicrobial prescribing interventions [79].

Vaccination programs and preventive healthcare strategies also contribute indirectly to antimicrobial stewardship by reducing the incidence and severity of infectious diseases that may otherwise require antimicrobial treatment. Similarly, optimization of nutrition, management of chronic diseases, parasite control, and early identification of infectious conditions represent preventive approaches that decrease reliance on antimicrobial therapy. Therefore, stewardship should be viewed not only as a prescribing-focused intervention but as part of a broader framework aimed at maintaining animal health and reducing avoidable antimicrobial exposure [73].

Finally, contemporary stewardship frameworks emphasize the importance of measuring program performance using predefined quality indicators. Evaluation may include quantitative measures such as antimicrobial prescribing rates and consumption metrics, as well as qualitative indicators including guideline adherence, appropriateness of antimicrobial selection, duration of therapy, frequency of culture submission, and clinical outcomes. Continuous monitoring of these indicators enables stewardship programs to identify areas requiring improvement and supports evidence-based refinement of future interventions [80].

The selection of appropriate quality indicators remains a critical challenge because antimicrobial consumption alone does not necessarily reflect prescribing quality. A reduction in antimicrobial use may represent improved prescribing practices, but it may also reflect reduced access to care, changes in case complexity, or unintended under-treatment. Consequently, comprehensive stewardship evaluation should combine quantitative indicators with measures of appropriateness, clinical outcomes, resistance trends, and patient safety parameters [80].

Potential stewardship indicators in companion animal medicine may include the proportion of cases receiving guideline-concordant therapy, frequency of culture and susceptibility testing when clinically indicated, use of highest-priority critically important antimicrobials, antimicrobial treatment duration, frequency of antimicrobial reassessment, and rates of antimicrobial de-escalation. Establishing standardized indicators would facilitate benchmarking between veterinary practices and improve the comparability of stewardship research across different healthcare systems [81].

Future development of veterinary antimicrobial stewardship programs will require greater integration of prescribing data, microbiological surveillance, and clinical outcome monitoring. Digital veterinary health records and automated data extraction systems may provide new opportunities for real-time assessment of prescribing behavior and identification of areas requiring intervention. However, successful implementation of such systems will depend on data quality, interoperability between platforms, clinician engagement, and appropriate interpretation of collected information [82].

Ultimately, effective antimicrobial stewardship in companion animal medicine requires a balance between scientific evidence, clinical judgment, and practical feasibility. Programs that combine guideline-based prescribing, diagnostic support, behavioral interventions, infection prevention strategies, surveillance, and continuous evaluation are most likely to achieve durable improvements. As antimicrobial resistance continues to evolve as a global One Health challenge, veterinary stewardship will remain an essential component of coordinated efforts to preserve antimicrobial efficacy for both animal and human health [82].

**Figure 1:**
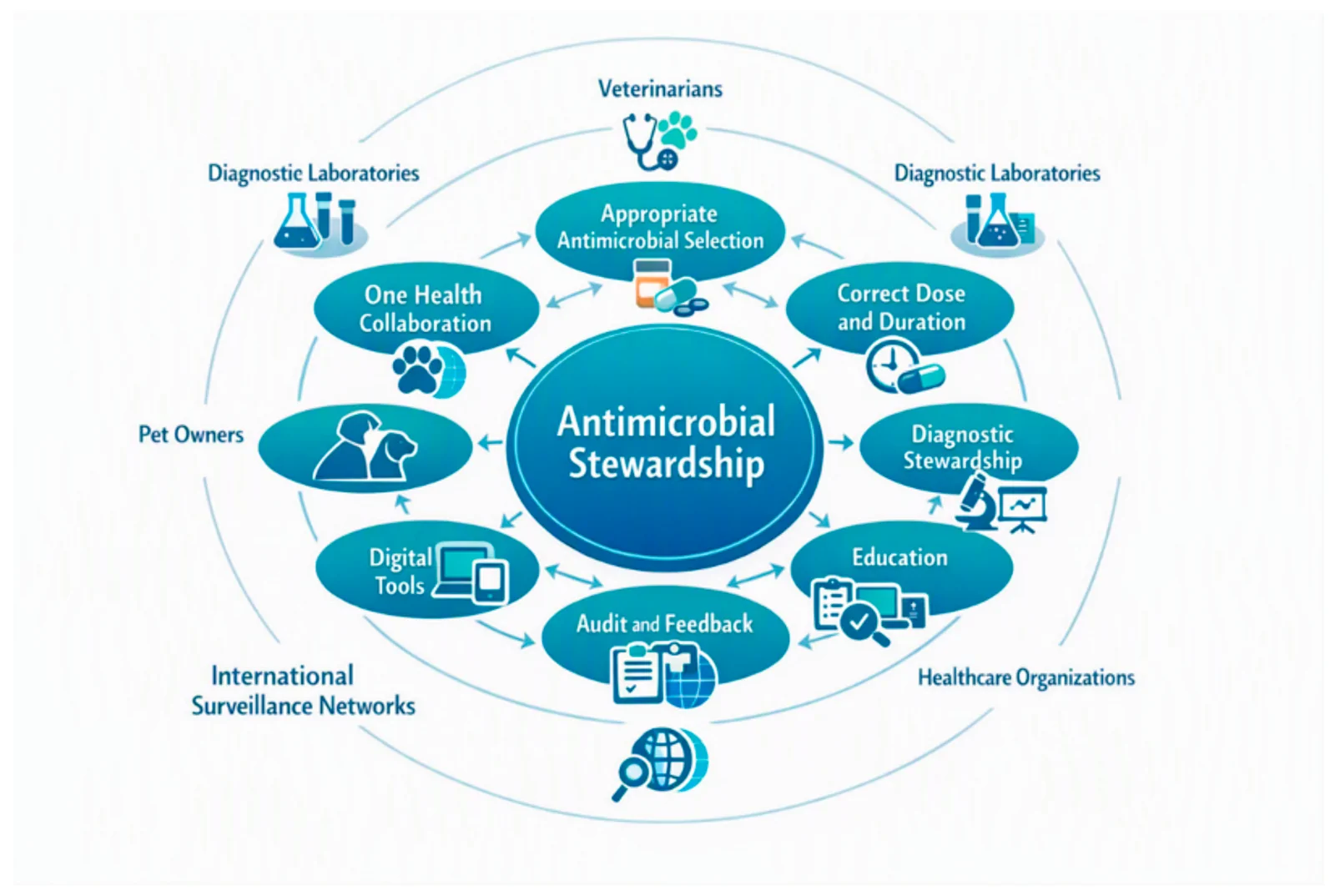
Conceptual framework of antimicrobial stewardship in companion animal medicine.

Figure 1 shows a conceptual framework illustrating the interconnected components of antimicrobial stewardship in companion animal medicine. The framework integrates antimicrobial prescribing decisions, diagnostic stewardship, behavioral determinants, organizational factors, surveillance systems, and One Health principles. Successful stewardship requires coordination between veterinarians, diagnostic laboratories, pet owners, healthcare organizations, and international surveillance networks.

## 4. Interventions to Reduce Antibiotic Prescribing

Although individual stewardship interventions have demonstrated measurable benefits, increasing evidence suggests that combining multiple complementary strategies produces greater and more sustainable improvements in prescribing quality than isolated interventions [83,84,85].

The effectiveness of stewardship interventions depends not only on the scientific validity of individual measures but also on their successful implementation within routine clinical practice. Interventions targeting multiple determinants of prescribing behavior—including knowledge, diagnostic capacity, organizational culture, clinician behavior, and institutional policies—are generally more successful than single-component approaches. Consequently, contemporary antimicrobial stewardship increasingly adopts multimodal implementation strategies capable of addressing both clinical and behavioral drivers of antimicrobial prescribing [83,84].

Selection of stewardship interventions should also consider local epidemiology, available diagnostic resources, practice size, patient population, and existing prescribing patterns. Tailoring interventions to local clinical needs may improve clinician engagement and increase the likelihood of sustained implementation [83,84].

Context-specific adaptation is particularly important in companion animal medicine because veterinary practices differ substantially in structure, resources, and patient populations. A stewardship intervention designed for a university referral hospital with specialist clinicians, microbiology facilities, and electronic prescribing systems may require substantial modification before implementation in smaller first-opinion practices. Successful programs should therefore maintain core stewardship principles while allowing flexibility in their practical application [83,84].

An additional consideration is the importance of intervention intensity and sustainability. Short-term educational campaigns may improve awareness temporarily but often produce limited long-term changes in prescribing behavior without reinforcement mechanisms. In contrast, interventions incorporating repeated feedback, continuous education, local leadership support, and monitoring of prescribing outcomes are more likely to generate durable improvements (Table 2) [83,84].

### 4.1. Audit and Feedback

Regular prescribing audits combined with individualized feedback allow clinicians to compare their prescribing behavior with established guidelines or peer benchmarks. Audit-feedback programs consistently demonstrate reductions in unnecessary antimicrobial prescribing [61,83].

Evidence from human healthcare consistently indicates that audit and feedback can improve antimicrobial prescribing, while emerging evidence from companion animal practice suggests that similar approaches may be effective in veterinary settings [51,52,61,62,63,64]. However, the magnitude and sustainability of these effects may differ between human and companion animal practice because of differences in clinical workflows, decision-making structures, and the veterinarian–owner relationship.

Evidence from both veterinary and human healthcare indicates that feedback is most effective when delivered regularly, benchmarked against peers, and accompanied by practical recommendations for improvement rather than simply presenting prescribing statistics [61,62].

The behavioral impact of audit and feedback is strongly influenced by the way information is presented. Feedback strategies incorporating social comparison, such as comparison with prescribing patterns of peers or institutional benchmarks, may promote behavioral change by increasing awareness of deviations from accepted practice norms. However, feedback should be designed carefully to avoid generating perceptions of criticism or surveillance, which may reduce clinician engagement. Supportive, confidential, and improvement-oriented feedback approaches are more likely to encourage sustained participation [61,62].

Audit and feedback interventions are particularly effective when feedback is timely, individualized, and based on objective prescribing indicators. Providing clinicians with comparative benchmarking against colleagues or institutional averages may increase awareness of prescribing habits and encourage voluntary behavioral change without imposing restrictive prescribing policies [63].

The selection of appropriate prescribing indicators is essential for meaningful audit-feedback programs. Measures may include overall antimicrobial prescription rates, use of highest-priority critically important antimicrobials, adherence to clinical guidelines, appropriateness of antimicrobial selection, treatment duration, frequency of diagnostic testing, and documentation of therapeutic justification. Combining quantitative prescribing metrics with qualitative assessments of appropriateness provides a more accurate evaluation of prescribing quality than consumption data alone [64].

In veterinary practice, audit-feedback interventions may be particularly valuable because prescribing decisions are often highly individualized and influenced by clinician experience. Regular review of prescribing data can identify areas of variation between clinicians, highlight opportunities for continuing education, and support the development of shared prescribing standards within practices. Importantly, the objective of these interventions is not to eliminate clinical judgment but to ensure that therapeutic decisions are consistent with current evidence whenever possible [86].

Longitudinal audit programs additionally enable veterinary practices to evaluate the sustainability of stewardship interventions, identify persistent prescribing challenges, and monitor improvements following implementation of educational or organizational initiatives. Continuous feedback cycles therefore represent an essential mechanism for maintaining long-term stewardship effectiveness [86].

Sustained effectiveness of audit-feedback programs requires integration into routine clinical workflows rather than implementation as isolated short-term projects. Regular feedback cycles, leadership support, and adaptation of interventions based on observed outcomes are necessary to maintain clinician engagement over time. Practices that combine audit-feedback with education, prescribing guidelines, diagnostic stewardship, and organizational support are more likely to achieve durable improvements compared with audit alone [86].

Emerging digital technologies may further enhance the scalability and efficiency of audit-feedback interventions. Automated extraction of prescribing data from electronic health records, real-time dashboards, and personalized prescribing reports may allow more frequent monitoring while reducing the administrative burden associated with traditional manual audits. However, successful implementation will depend on data accuracy, interoperability of veterinary software systems, and clinician acceptance of digital monitoring tools [88].

### 4.2. Clinical Decision Support Systems

Electronic prescribing systems, treatment algorithms, and clinical decision-support tools assist veterinarians in selecting appropriate antimicrobial therapy according to current guidelines and patient-specific information [50].

Clinical decision-support systems (CDSS) represent an emerging component of antimicrobial stewardship because they have the potential to translate complex clinical evidence into accessible recommendations at the point of care. By providing structured guidance during the prescribing process, these systems may reduce unnecessary variation between clinicians, improve adherence to antimicrobial guidelines, and support more consistent therapeutic decisions. In companion animal medicine, where prescribing practices may vary substantially between individual veterinarians and clinical settings, decision-support technologies offer an opportunity to promote evidence-based standardization while preserving clinician autonomy [50].

The incorporation of artificial intelligence and machine learning algorithms into clinical decision-support systems may further improve prescribing accuracy by integrating patient history, laboratory results, local resistance patterns, and previous treatment responses into individualized therapeutic recommendations [50].

Artificial intelligence-based approaches may substantially expand the capabilities of future stewardship systems by identifying complex relationships between clinical variables that are difficult to recognize through conventional decision-making processes. Machine learning models could potentially assist in predicting the probability of bacterial infection, estimating antimicrobial resistance risk, identifying patients suitable for antimicrobial de-escalation, and recommending treatment options based on individual patient characteristics and local epidemiological data. However, these technologies require extensive validation using high-quality veterinary datasets before widespread clinical adoption [50].

Clinical decision-support systems may also reduce prescribing variability by standardizing therapeutic recommendations across clinicians while simultaneously decreasing reliance on memory alone. Automated alerts identifying prolonged treatment duration, inappropriate antimicrobial selection, duplicate therapy, or deviations from established prescribing guidelines may further support evidence-based clinical decision-making [65].

The effectiveness of electronic stewardship tools depends strongly on their integration into clinical workflow. Systems that require additional documentation, interrupt consultations excessively, or generate frequent non-actionable alerts may lead to alert fatigue and reduced clinician engagement. Therefore, successful CDSS implementation requires careful design principles, including user-centered development, clinically relevant alerts, minimal disruption of workflow, and continuous refinement based on user feedback [66].

Another important consideration is the balance between standardization and individualized clinical judgment. Decision-support systems should function as tools that assist rather than replace veterinary expertise, particularly because companion animal patients often present with complex combinations of comorbidities, owner preferences, diagnostic limitations, and variable disease severity. The most effective systems are likely to combine evidence-based recommendations with flexibility that allows clinicians to adapt treatment decisions according to specific patient circumstances [66].

Successful implementation of digital stewardship tools nevertheless depends on clinician acceptance, integration into existing clinical workflows, ease of use, and regular updating of embedded recommendations to reflect evolving clinical evidence and antimicrobial resistance patterns. Poorly designed electronic systems may increase workload and reduce user adherence despite their theoretical benefits [66].

Regular updating of clinical decision-support systems is essential because antimicrobial recommendations may become outdated as resistance patterns, treatment guidelines, and available evidence evolve. Integration with antimicrobial resistance surveillance databases and institutional prescribing data may allow future systems to provide dynamic recommendations reflecting local epidemiology rather than relying solely on static guidelines [55].

Data governance and interoperability represent additional challenges for implementation. Veterinary electronic health record systems vary considerably between practices, and standardized approaches for collecting, sharing, and analyzing prescribing data remain limited. Development of interoperable digital infrastructures will be necessary to fully realize the potential of electronic stewardship tools and enable large-scale surveillance of antimicrobial use and resistance trends [87,89].

Future advances may involve integration of CDSS with rapid diagnostic technologies, microbiological databases, and automated surveillance platforms, creating comprehensive decision-support ecosystems capable of assisting clinicians throughout the entire antimicrobial prescribing pathway—from initial diagnostic assessment to treatment reassessment and de-escalation. Such integrated systems represent a promising direction for improving antimicrobial stewardship while maintaining efficient clinical workflows [87,89].

### 4.3. Multifaceted Stewardship Programs

Programs combining multiple interventions—including education, guidelines, diagnostic stewardship, audits, and feedback—have generally shown greater effectiveness than isolated measures [90].

From an implementation perspective, successful multifaceted stewardship programs require careful planning, stakeholder engagement, identification of local barriers, and continuous evaluation of outcomes. The effectiveness of these programs depends not only on which interventions are included but also on how they are delivered, who implements them, and whether they are compatible with routine clinical workflows. Frameworks derived from implementation science can help veterinary practices identify determinants of success and adapt stewardship strategies to different healthcare environments [89].

Multifaceted stewardship programs frequently combine clinician education, locally adapted prescribing guidelines, prospective audit and feedback, diagnostic stewardship, electronic decision-support systems, antimicrobial consumption surveillance, and institutional leadership support. Addressing multiple determinants of prescribing behavior simultaneously increases the likelihood of sustained improvements compared with interventions targeting a single aspect of antimicrobial use [91]. In resource-limited first-opinion practices, these programs can be implemented using relatively simple measures such as locally adapted prescribing guidelines, brief case-based education, periodic peer review or audit-feedback, selective diagnostic testing for higher-risk cases, and standardized communication materials for owners, without requiring dedicated stewardship personnel or sophisticated digital infrastructure.

The combination of complementary interventions may also generate synergistic effects. For example, prescribing guidelines provide evidence-based recommendations, while audit and feedback increase awareness of individual prescribing patterns, diagnostic stewardship reduces uncertainty, and electronic tools facilitate access to relevant information during clinical decision-making. When these components are integrated within a supportive organizational culture, they can reinforce each other and promote long-term changes in prescribing habits [89].

Implementation strategies should also include periodic reassessment of program effectiveness using predefined quality indicators. Continuous monitoring allows stewardship teams to identify unsuccessful interventions, refine implementation strategies, and respond to changing antimicrobial resistance patterns or evolving clinical evidence. This iterative quality-improvement approach is increasingly recognized as a defining characteristic of successful stewardship programs [92].

Adaptability is particularly important in companion animal medicine because veterinary healthcare systems vary substantially between countries and practice types. A successful stewardship model should therefore preserve core principles—appropriate antimicrobial selection, optimized dosing, diagnostic support, reassessment of therapy, and resistance monitoring—while allowing flexibility in implementation according to local circumstances. This approach increases the likelihood of adoption and sustainability across diverse clinical settings [92].

Ultimately, the long-term success of multifaceted stewardship programs will depend on their ability to become embedded within routine veterinary practice rather than functioning as temporary research initiatives. Sustainable programs require institutional commitment, continuous education, reliable data systems, and regular adaptation to emerging scientific evidence [92].

## 5. Evidence of Effectiveness

Most published studies report reductions in overall antibiotic prescribing following implementation of stewardship interventions, although the predominance of observational and before-and-after study designs limits causal interpretation. Several investigations also report decreased use of critically important antimicrobials alongside favorable patient outcomes. Overall, current evidence suggests that stewardship interventions may improve prescribing practices without adversely affecting short-term treatment outcomes, but the strength of the available causal evidence remains limited.

The observed reductions in antimicrobial prescribing across veterinary studies suggest that stewardship interventions can modify clinical behavior when appropriately designed and implemented. However, the interpretation of effectiveness requires consideration of whether reductions represent improved prescribing quality rather than simple decreases in antimicrobial availability. Successful stewardship should achieve optimization of therapy by reducing unnecessary exposure while ensuring that patients with confirmed or suspected bacterial infections continue to receive timely and appropriate treatment [39,51].

Collectively, available studies indicate that stewardship interventions are capable of improving not only the quantity but also the quality of antimicrobial prescribing. Increasingly, investigators emphasize appropriate antimicrobial selection, adherence to prescribing guidelines, optimization of treatment duration, and greater use of microbiological diagnostics as complementary indicators of stewardship success. These qualitative improvements may ultimately be more clinically meaningful than reductions in antimicrobial consumption alone, as they better reflect evidence-based prescribing practices [39,51].

**Figure 2:**
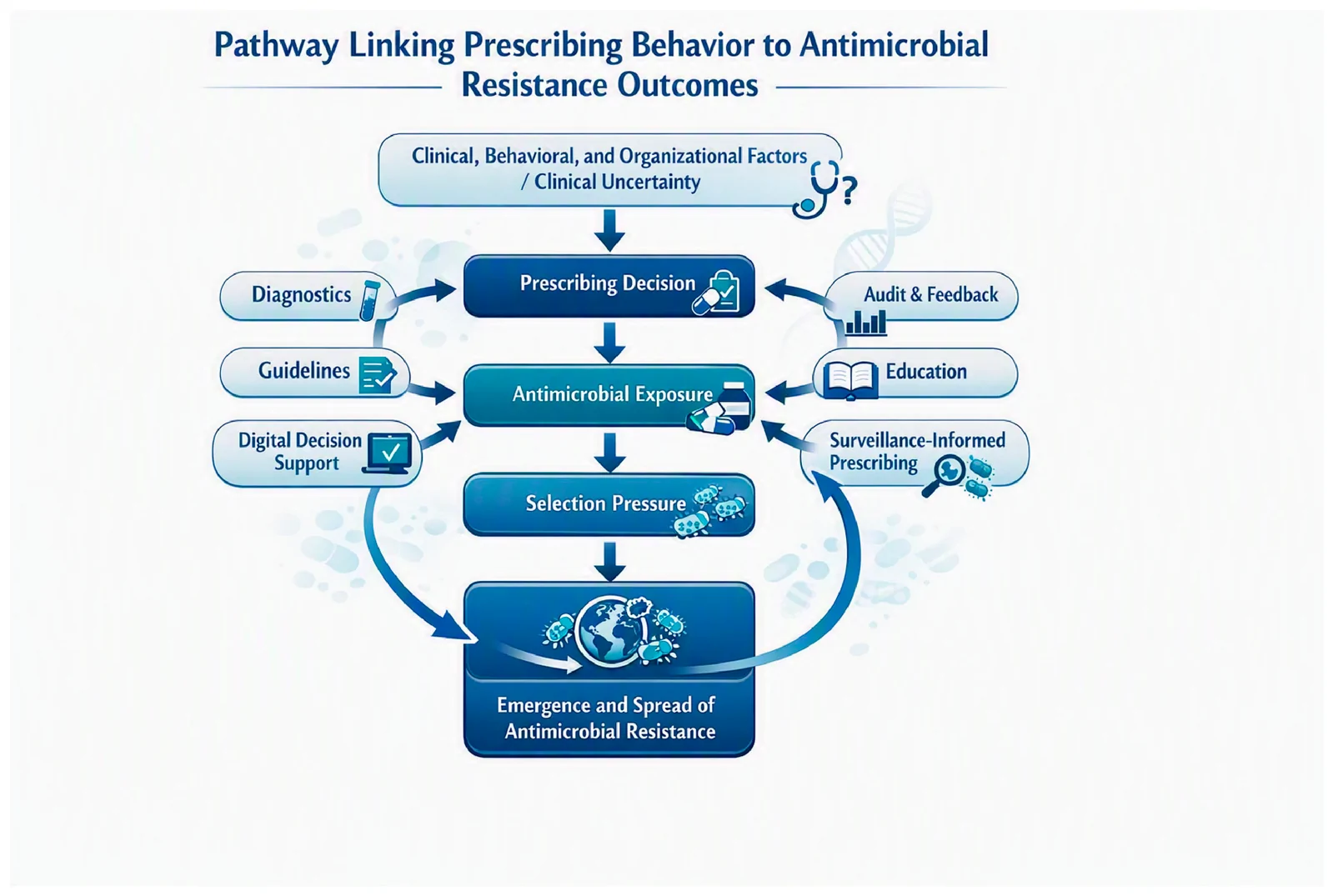
Pathway linking prescribing behavior to antimicrobial resistance outcomes.

Figure 2 shows a proposed pathway illustrating how clinical, behavioral, and organizational factors influence antimicrobial prescribing and ultimately affect antimicrobial resistance development. Stewardship interventions act by modifying prescribing behavior, reducing unnecessary antimicrobial exposure, and supporting preservation of antimicrobial efficacy.

This distinction between antimicrobial quantity and prescribing quality is particularly important in companion animal medicine, where clinical decisions are frequently individualized and influenced by patient-specific factors. A successful stewardship intervention may not necessarily produce the greatest reduction in antimicrobial use but rather the greatest improvement in therapeutic appropriateness, including better drug selection, optimized treatment duration, and improved diagnostic justification [51].

Improved clinician confidence represents an important but often underestimated outcome of stewardship interventions. Fear of treatment failure, concern regarding disease progression, and uncertainty during initial clinical assessment are recognized barriers to reducing unnecessary antimicrobial use. Educational programs, decision-support tools, and feedback interventions may therefore have effects beyond prescribing frequency by changing clinicians’ perceptions of risk and increasing acceptance of evidence-based non-antimicrobial management strategies [93].

Nevertheless, interpretation of these findings should be undertaken cautiously because much of the current evidence is derived from before-and-after observational studies that remain susceptible to selection bias, confounding factors, and temporal changes unrelated to stewardship interventions. High-quality randomized controlled trials remain relatively scarce in veterinary medicine.

The limited availability of randomized controlled trials represents a major challenge for determining the causal effectiveness of veterinary stewardship interventions. While observational studies provide valuable insights into real-world implementation, they may overestimate intervention effects because participating practices or clinicians may already be more motivated toward responsible antimicrobial use. Future research should increasingly incorporate robust study designs, including multicenter controlled trials, interrupted time-series analyses, and pragmatic implementation studies conducted under routine clinical conditions.

The predominance of observational study designs also limits the ability to establish causal relationships between stewardship interventions and observed improvements in prescribing behavior. Changes may partly reflect increasing awareness of antimicrobial resistance, evolving clinical guidelines, or concurrent institutional initiatives rather than stewardship interventions alone. Furthermore, heterogeneity in study populations, intervention design, follow-up duration, and outcome definitions complicates direct comparison between published investigations and limits opportunities for quantitative evidence synthesis [52].

Clinical outcomes reported across studies generally include similar cure rates, low recurrence rates, and no increase in complications despite reduced antimicrobial prescribing [94].

Importantly, available studies generally report no clear deterioration in short-term clinical outcomes following reductions in antimicrobial prescribing, although the evidence remains limited by short follow-up periods, predominantly observational study designs, and the frequent inclusion of motivated practices or referral settings. These findings provide some reassurance regarding the clinical safety of stewardship interventions, but longer-term and more broadly representative studies are needed to confirm that antimicrobial optimization can be achieved without compromising patient outcomes [94].

Maintaining clinical outcomes while reducing unnecessary antimicrobial exposure represents one of the strongest indicators of stewardship success. However, future studies should expand outcome assessment beyond short-term treatment success and include longer-term measures such as recurrence rates, antimicrobial resistance development, quality of life, adverse drug reactions, and owner satisfaction. These broader outcomes would provide a more comprehensive understanding of the clinical impact of stewardship interventions [93].

Development of standardized appropriateness assessment tools remains an important research priority. Currently, substantial variation exists in how studies define appropriate antimicrobial use, limiting comparison between investigations. Validated assessment frameworks incorporating clinical indication, antimicrobial selection, dosing regimen, duration, diagnostic support, and patient outcome measures would improve consistency and facilitate evaluation of stewardship programs across different veterinary settings [95].

Economic evaluations suggest that stewardship programs may also decrease treatment costs through more appropriate antimicrobial selection and reduced unnecessary prescriptions [95].

The economic impact of antimicrobial stewardship extends beyond immediate reductions in medication costs. Improved prescribing practices may reduce adverse drug reactions, unnecessary diagnostic procedures, treatment failures associated with inappropriate antimicrobial selection, and future healthcare costs related to antimicrobial-resistant infections. Therefore, economic evaluations should consider both direct and indirect consequences of stewardship implementation [95].

However, comprehensive cost-effectiveness analyses remain limited, particularly regarding long-term financial benefits associated with reduced antimicrobial resistance and decreased hospitalization resulting from resistant infections [95].

The limited availability of veterinary health-economic studies represents a significant knowledge gap. Many potential benefits of stewardship, including preservation of antimicrobial efficacy, prevention of resistant infections, and reduction in future treatment complexity, are difficult to quantify using traditional economic models. Future studies should incorporate broader cost-effectiveness frameworks capable of capturing both immediate clinical benefits and long-term population-level impacts.

Potential economic benefits may also arise from fewer adverse drug reactions, reduced diagnostic uncertainty through more targeted testing strategies, shorter treatment courses, and decreased use of expensive broad-spectrum antimicrobials. Nevertheless, these potential savings remain insufficiently quantified within veterinary medicine, highlighting an important area for future health-economic research [95].

Economic analyses should also consider the resources required to implement stewardship programs, including clinician training, diagnostic infrastructure, digital systems, and personnel time. Although these investments may initially increase operational costs, they may provide substantial long-term value by improving healthcare efficiency, reducing unnecessary antimicrobial exposure, and supporting sustainable clinical practice [41].

Another important limitation of the current evidence is the relatively short duration of follow-up in many published studies. Sustained reductions in antimicrobial prescribing over several years have been evaluated less frequently, making it difficult to determine whether behavioral improvements remain stable after initial stewardship implementation. Long-term prospective studies are therefore needed to assess the durability of stewardship interventions and their continued effectiveness under routine clinical conditions [41].

Long-term sustainability represents one of the greatest challenges for antimicrobial stewardship programs. Initial improvements may decline when educational campaigns end, feedback becomes less frequent, or organizational support decreases. Sustainable stewardship therefore requires integration into routine clinical governance, ongoing monitoring, regular feedback mechanisms, and continuous adaptation to changing clinical circumstances [96].

Perhaps the greatest unresolved question concerns the direct relationship between stewardship interventions and antimicrobial resistance itself. Although reductions in antimicrobial consumption are generally assumed to decrease selective pressure for resistant bacteria, relatively few veterinary studies have demonstrated measurable reductions in antimicrobial resistance following stewardship implementation. Such investigations require prolonged surveillance, standardized microbiological methodologies, and large multicenter datasets capable of detecting gradual ecological changes in bacterial populations. Consequently, establishing a direct causal link between stewardship and reductions in antimicrobial resistance remains an important research priority [41].

The relationship between antimicrobial stewardship and resistance outcomes is particularly complex because antimicrobial resistance is influenced by multiple interacting factors, including antimicrobial exposure, bacterial population dynamics, infection prevention practices, environmental contamination, animal movement, and transmission between animal and human populations. Consequently, demonstrating measurable reductions in resistance following stewardship interventions requires long-term integrated surveillance approaches rather than short-term prescribing evaluations alone [96].

Future research should increasingly combine antimicrobial prescribing data with microbiological surveillance, molecular epidemiology, and genomic approaches to determine whether stewardship interventions influence resistance patterns at the population level. Integration of veterinary, human, and environmental surveillance within a One Health framework may provide a more complete understanding of how antimicrobial optimization contributes to resistance mitigation across interconnected ecosystems (Table 3) [96].

Overall, the strength and directness of evidence supporting antimicrobial stewardship interventions vary across clinical settings. Evidence from companion animal practice provides direct support for interventions such as prescribing guidelines, audit and feedback, education, and multifaceted stewardship programs [39,51,52,67,82]. In contrast, evidence supporting some behavioural, organisational, diagnostic, and digital strategies remains predominantly derived from human healthcare or broader veterinary settings [50,56,57,58,59,60,61,62,63,64,65,66,87,88]. These findings should therefore be interpreted as transferable evidence rather than direct evidence of effectiveness in companion animal practice. Extrapolation from human healthcare may be justified when veterinary evidence is limited, particularly for interventions based on general principles of behaviour change, implementation science, or clinical decision support; however, differences in clinical context, owner involvement, financial considerations, regulatory frameworks, and healthcare organisation may limit transferability. Further companion-animal-specific research is therefore required to determine whether interventions demonstrated in human healthcare produce comparable effects in veterinary practice.

## 6. Challenges and Knowledge Gaps

Although stewardship interventions show considerable promise, several limitations remain. Most available studies are observational, involve relatively small sample sizes, and differ substantially in methodology, making direct comparisons difficult. Standardized outcome measures and multicenter randomized studies are still limited. Additional research is needed to evaluate the long-term sustainability and cost-effectiveness of stewardship interventions [97].

A major challenge in advancing veterinary antimicrobial stewardship research is the limited methodological consistency between studies. Differences in study design, intervention components, antimicrobial definitions, prescribing indicators, and outcome measurements make it difficult to determine which strategies are most effective under specific clinical conditions. Establishing standardized methodologies and internationally accepted reporting frameworks would improve evidence synthesis and support more reliable comparisons between stewardship programs [97].

The complexity of stewardship interventions also creates challenges in identifying the individual components responsible for observed improvements. Multifaceted programs often combine education, guidelines, audits, diagnostics, and organizational changes, making it difficult to determine which elements contribute most strongly to behavioral change. Future research using implementation science approaches, including process evaluations and mixed-methods designs, may help clarify the mechanisms underlying successful stewardship interventions [89].

Developing validated prescribing appropriateness assessment tools should therefore represent a priority for veterinary stewardship research. Such tools should incorporate clinical indication, antimicrobial choice, dose optimization, treatment duration, diagnostic support, and patient outcomes while remaining practical for use in routine clinical environments. Standardized assessment approaches would facilitate benchmarking between practices and strengthen the evidence supporting stewardship interventions [98].

Moreover, relatively little evidence is available regarding stewardship implementation in first-opinion veterinary practices, where the majority of antimicrobial prescriptions are generated. Future research should therefore prioritize pragmatic multicenter studies reflecting routine clinical practice rather than referral hospital settings alone [98].

Further knowledge gaps remain regarding the influence of geographical variation, antimicrobial availability, regulatory policies, socioeconomic factors, and differences in veterinary healthcare systems on stewardship implementation. These contextual variables may substantially influence prescribing behavior and should be considered when interpreting study findings or transferring stewardship interventions between countries [98].

Limited information is also available regarding owner-related outcomes, clinician acceptance of stewardship interventions, and barriers affecting long-term adherence to prescribing recommendations. Understanding these behavioral and organizational determinants will be essential for designing stewardship programs capable of achieving sustainable improvements in routine veterinary practice [98].

Another important knowledge gap concerns the role of emerging technologies in supporting stewardship implementation. Although artificial intelligence, electronic prescribing systems, and automated surveillance platforms show considerable potential, their effectiveness, safety, cost-effectiveness, and acceptability in veterinary practice remain insufficiently evaluated. Future studies should assess not only technological performance but also practical implementation challenges and their impact on clinician behavior and patient outcomes [98].

Finally, relatively few studies have evaluated microbiological outcomes alongside prescribing outcomes. Future investigations should increasingly integrate antimicrobial resistance surveillance, molecular epidemiology, and genomic characterization of resistant pathogens to better understand the ecological impact of stewardship interventions on bacterial populations over time (Table 4) [98].

## 7. Future Perspectives

Future antimicrobial stewardship programs are likely to increasingly integrate technological innovation, behavioral strategies, and organizational approaches to support evidence-based prescribing. Key future directions include the use of artificial intelligence, electronic prescribing systems, rapid molecular diagnostics, point-of-care testing, integrated surveillance, and implementation strategies adapted to routine companion animal practice.

The future development of antimicrobial stewardship in companion animal medicine will likely depend on the successful integration of technological innovation with behavioral and organizational strategies. Although digital tools have considerable potential to improve prescribing decisions, technology alone is unlikely to achieve sustained improvements without addressing the human factors that influence antimicrobial use. Future stewardship models will therefore need to combine advanced analytics with clinician education, communication strategies, workflow optimization, and institutional support [99].

Continued advances in artificial intelligence may facilitate predictive clinical decision-support systems capable of estimating the probability of bacterial infection, recommending individualized antimicrobial therapy, identifying patients suitable for antimicrobial de-escalation, and automatically detecting prescribing patterns requiring stewardship review. As these technologies mature, careful clinical validation and appropriate regulatory oversight will remain essential to ensure safe implementation in veterinary medicine [99].

Artificial intelligence-based stewardship systems may eventually support real-time clinical decision-making by integrating multiple sources of information, including electronic health records, laboratory findings, antimicrobial susceptibility data, patient history, and local resistance patterns. Predictive models may assist clinicians in identifying patients at increased risk of resistant infections, selecting appropriate empirical therapy, and recognizing opportunities for antimicrobial discontinuation or de-escalation. However, implementation should be accompanied by rigorous validation, transparency of algorithmic decision-making, and continuous monitoring to prevent inappropriate recommendations or unintended clinical consequences [100].

An important future challenge will be ensuring that artificial intelligence systems are developed using representative veterinary datasets. Many current machine-learning approaches are limited by incomplete data, inconsistent electronic records, and underrepresentation of diverse patient populations. Collaborative efforts to create large, high-quality veterinary datasets will be essential for developing reliable and generalizable AI-based stewardship tools [101].

The increasing availability of electronic health records offers opportunities to develop real-time antimicrobial surveillance systems capable of monitoring prescribing trends, identifying inappropriate prescribing patterns, and providing automated stewardship feedback to clinicians [101].

Integration of electronic prescribing data with microbiological laboratory databases may further enable near real-time surveillance of antimicrobial resistance, allowing stewardship teams to rapidly identify emerging resistance patterns and adapt prescribing recommendations accordingly. Such integrated digital infrastructure represents one of the most promising developments for future stewardship programs [101].

A One Health approach will remain central to future antimicrobial stewardship because antimicrobial resistance does not respect species or environmental boundaries. Companion animals share close contact with humans and may participate in the exchange of resistant microorganisms and resistance genes within households and communities. Strengthening collaboration between veterinary, medical, environmental, and public health sectors will therefore be essential for developing coordinated strategies capable of addressing antimicrobial resistance at a global scale [100].

Future stewardship initiatives should also strengthen collaboration between veterinary and human healthcare sectors to improve integrated surveillance of antimicrobial resistance, facilitate data sharing, and develop coordinated interventions targeting shared resistant pathogens [100].

International collaboration may additionally promote harmonization of antimicrobial prescribing guidelines, standardized stewardship indicators, collaborative multicenter research, and shared educational resources for veterinary professionals. Coordinated global surveillance initiatives will improve understanding of antimicrobial resistance epidemiology while facilitating evidence-based policy development across different healthcare sectors [98].

Future research should also explore implementation science methodologies capable of identifying the most effective strategies for translating stewardship recommendations into routine clinical practice. Combining technological innovation with behavioral interventions, organizational change, and continuous quality improvement may provide the greatest opportunity for achieving durable reductions in unnecessary antimicrobial prescribing [98].

Ultimately, the future of veterinary antimicrobial stewardship will depend on creating adaptive systems capable of continuously learning from clinical data, scientific advances, and changing resistance patterns. Integration of digital technologies, behavioral science, surveillance networks, and One Health collaboration offers the greatest potential for developing sustainable stewardship models that protect antimicrobial efficacy while maintaining high standards of patient care [98].

## 8. Materials and Methods

This article was designed as a narrative review synthesizing current evidence on antimicrobial stewardship in companion animal medicine. The review focused on antimicrobial prescribing behavior, diagnostic stewardship, behavioral and organizational determinants of prescribing, audit and feedback, digital decision-support tools, surveillance systems, One Health approaches, and implementation challenges relevant to small animal veterinary practice.

A structured literature search was performed using PubMed, Scopus, Web of Science, Google Scholar, and relevant organizational or professional websites. Search terms included combinations of “antimicrobial stewardship”, “antibiotic prescribing”, “companion animals”, “small animal practice”, “veterinary medicine”, “diagnostic stewardship”, “audit and feedback”, “clinical decision support”, “antimicrobial resistance surveillance”, “One Health”, “dogs”, and “cats”. The main literature search was completed on 31 July 2026, followed by targeted searches for additional relevant studies during manuscript preparation through 3 August 2026. Additional records were identified through reference lists of relevant reviews, guidelines, and key articles. The searches identified 2847 records across the selected databases and additional sources. After removal of duplicates, 2214 records remained for screening.

Eligibility criteria were defined to improve transparency and consistency in the selection of literature. Publications were considered eligible when they addressed antimicrobial use, antimicrobial stewardship interventions, diagnostic stewardship, antimicrobial resistance, prescribing behavior, or implementation strategies in veterinary medicine, with particular relevance to companion animals and small animal practice. Studies conducted in food-producing animals or human healthcare were also considered when they provided transferable evidence regarding stewardship principles, behavioral or organizational interventions, surveillance approaches, or One Health strategies. Peer-reviewed original studies, systematic and scoping reviews, clinical guidelines, and relevant policy or professional documents were considered. Priority was given to publications providing empirical evidence on antimicrobial prescribing, stewardship interventions, diagnostic practices, clinical outcomes, antimicrobial resistance, or implementation processes.

**Figure 3:**
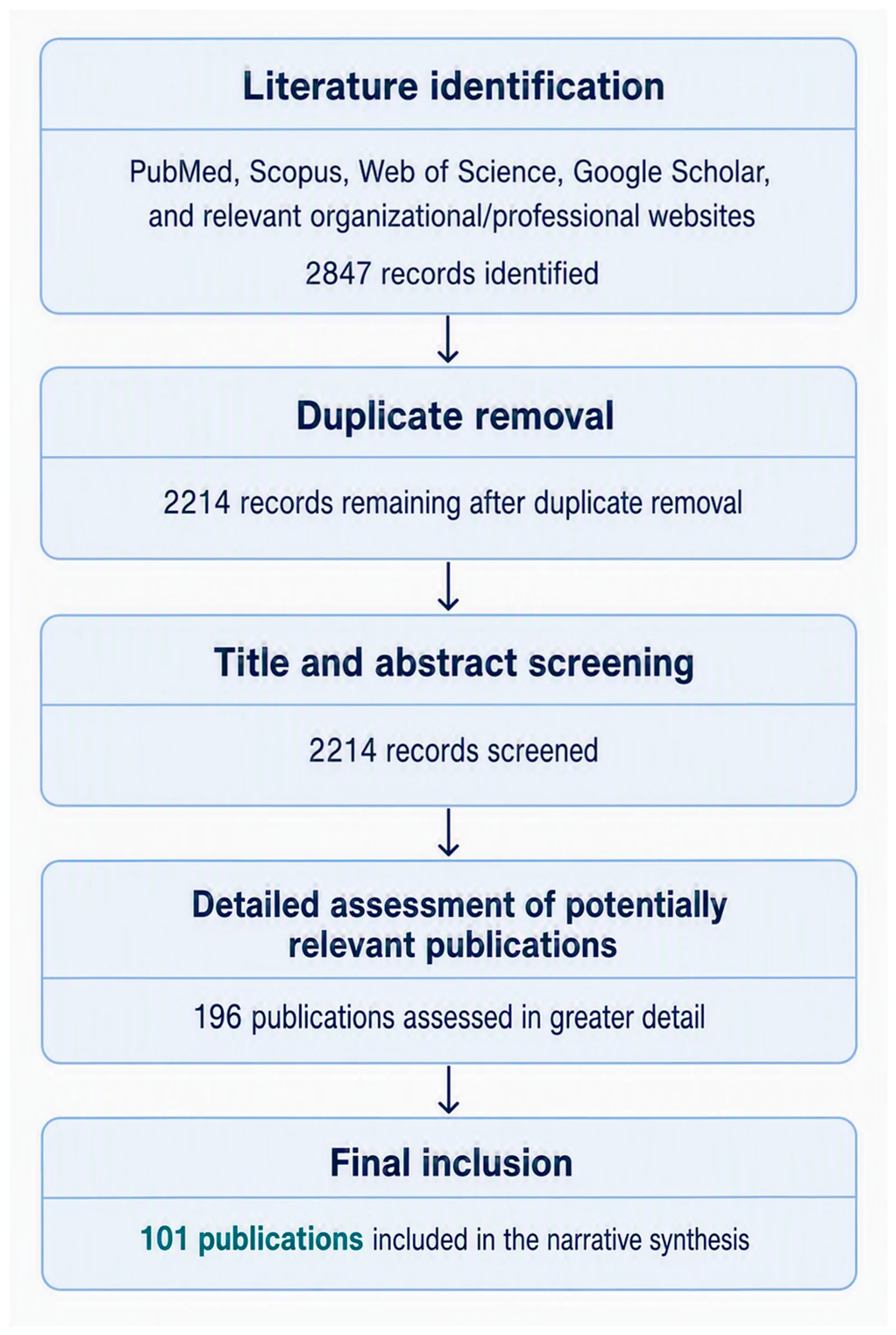
Overview of the literature identification, screening, assessment, and inclusion process used for the narrative review.

Publications were excluded when they were unrelated to antimicrobial use or stewardship, focused exclusively on topics without relevance to veterinary or One Health antimicrobial stewardship, or did not provide sufficient information to contribute to the objectives of the review. Duplicate records were removed before screening. The literature selection process involved an initial screening of titles and abstracts, followed by assessment of potentially relevant publications based on their relevance to the review objectives and thematic scope. Following title and abstract screening, 196 publications were considered potentially relevant and assessed in greater detail. Ultimately, 101 publications were included in the narrative synthesis. The overall literature review process is summarized in Figure 3.

The search prioritized peer-reviewed articles published in English that addressed antimicrobial use, prescribing behavior, stewardship interventions, antimicrobial resistance, or implementation strategies in veterinary medicine, with particular emphasis on companion animal practice. Relevant studies from human healthcare and broader One Health literature were also considered when they provided conceptual, methodological, or implementation insights applicable to veterinary stewardship. Importantly, evidence derived from human healthcare was used primarily to provide conceptual or methodological context and was not considered equivalent to evidence generated in companion animal practice. Throughout the synthesis, findings from companion animal studies were prioritized when available, whereas evidence from human medicine or other veterinary sectors was used to inform transferable concepts, such as behavioral determinants, implementation strategies, audit and feedback, and clinical decision support. Studies conducted in food-producing animals were considered separately because their prescribing context, population-level treatment patterns, economic drivers, and regulatory environment differ substantially from those of companion animal practice. Guidelines and policy documents from international or professional organizations were included when directly relevant to antimicrobial prescribing or stewardship practice.

Publications were selected based on their relevance to the objectives of the review, methodological quality, contribution to understanding stewardship interventions, and applicability to companion animal medicine. Priority was given to systematic reviews, scoping reviews, clinical guidelines, observational studies, implementation studies, intervention trials, and studies reporting prescribing outcomes, diagnostic practices, antimicrobial resistance trends, or behavioral determinants of antimicrobial use.

Because this was designed as a narrative review rather than a systematic review, selection was not based exclusively on predefined quantitative eligibility scores. Final inclusion was determined according to relevance, methodological contribution, topical coverage, and applicability to companion animal medicine. This approach allowed integration of evidence from veterinary, human-health, and One Health literature while acknowledging the potential for selection bias inherent in narrative evidence synthesis.

Conflicting findings were considered in the narrative synthesis by comparing study design, methodological characteristics, clinical setting, population, and consistency of reported outcomes. Where evidence was inconsistent, greater weight was given to higher-quality studies and findings supported by multiple independent sources. No formal standardized quality appraisal tool was applied because the review was designed as a narrative rather than systematic review; methodological limitations and potential sources of bias were nevertheless considered when interpreting the evidence.

Data were extracted narratively and organized into thematic categories reflecting the major components of antimicrobial stewardship: determinants of prescribing, stewardship frameworks, diagnostic stewardship, audit and feedback, clinical decision-support systems, multifaceted interventions, evidence of effectiveness, knowledge gaps, and future perspectives. Because of heterogeneity in study designs, clinical settings, intervention types, and outcome measures, no quantitative meta-analysis was performed.

The review followed principles commonly recommended for transparent evidence synthesis, including clear reporting of information sources, search concepts, eligibility considerations, and synthesis approach. However, it was not registered as a systematic review protocol and did not aim to provide an exhaustive quantitative assessment of all available evidence. Instead, the objective was to provide a critical and practice-oriented synthesis of current knowledge relevant to antimicrobial stewardship implementation in companion animal medicine.

## 9. Conclusions

Antimicrobial stewardship interventions represent effective strategies for reducing unnecessary antibiotic prescribing in small animal veterinary practice. Evidence from companion animal practice indicates that clinical guidelines, education, audit and feedback, and multifaceted stewardship programs can improve prescribing quality without apparent adverse effects on patient outcomes, although the strength of evidence varies among intervention types. Evidence from human healthcare and other veterinary sectors provides additional support for several stewardship approaches, but these findings should be interpreted as complementary and potentially transferable rather than direct evidence for companion animal practice.

Where evidence from human healthcare is used to inform recommendations for companion animal practice, extrapolation is based on shared principles of antimicrobial stewardship and behaviour change; however, differences in the veterinarian–owner–animal relationship, financial considerations, diagnostic availability, clinical workflows, and regulatory contexts may limit transferability. Companion-animal-specific validation is therefore required before human-derived interventions can be considered broadly applicable to veterinary practice.

In clinical practice, antimicrobial stewardship can be initiated as a stepwise quality-improvement process adapted to the resources and workflow of each veterinary practice. A practical starting point is to review current antimicrobial prescribing patterns and identify priority areas for improvement, such as unnecessary antimicrobial use, prolonged treatment duration, use of highest-priority critically important antimicrobials, or insufficient diagnostic investigation. Practices can then designate a stewardship lead or champion, develop or adapt concise local prescribing guidelines for common conditions, establish clear criteria for diagnostic testing and treatment reassessment, and provide targeted education for veterinarians and support staff. These measures should be integrated into routine clinical workflows and supported by clear communication with pet owners regarding diagnostic uncertainty, treatment expectations, and alternatives to antimicrobial therapy. Regular audit and supportive feedback can subsequently be used to monitor prescribing quality and identify further opportunities for improvement. Smaller first-opinion practices can begin with a limited number of feasible interventions, such as local prescribing guidance, targeted diagnostic testing, treatment-duration review, and periodic case-based audit, whereas larger practices may additionally implement electronic decision-support systems, automated surveillance, and formal stewardship teams. Repeated assessment of predefined prescribing and clinical indicators can then guide refinement and progressive expansion of the stewardship program.

Current evidence indicates that stewardship should not be viewed solely as a strategy for reducing antimicrobial consumption but rather as a comprehensive quality-improvement framework that promotes evidence-based clinical decision-making while safeguarding antimicrobial efficacy for future generations.

Continued investment in research, education, and implementation strategies will be essential to ensure sustainable improvements in antimicrobial use and to mitigate the growing threat of antimicrobial resistance.

Future progress will depend on the integration of behavioral science, implementation research, advanced diagnostics, digital health technologies, and international collaboration to establish sustainable antimicrobial stewardship programs capable of responding to the evolving challenges posed by antimicrobial resistance.

Despite encouraging progress, antimicrobial stewardship should be regarded as a dynamic and continuously evolving discipline that requires regular adaptation to emerging scientific evidence, changing resistance patterns, and advances in diagnostic and digital technologies. Long-term success will depend not only on the development of innovative stewardship interventions but also on their effective implementation, continuous evaluation, and integration into everyday veterinary clinical practice.

Strengthening collaboration among clinicians, researchers, microbiologists, professional organizations, policymakers, and pet owners will be fundamental for ensuring that stewardship initiatives achieve meaningful and sustainable improvements in antimicrobial prescribing. Continued investment in high-quality research, standardized surveillance systems, and international cooperation will further strengthen the evidence base supporting veterinary antimicrobial stewardship while contributing to the global One Health response to antimicrobial resistance.

## Figures and Tables

**Table 2:** Antimicrobial stewardship interventions in companion animal medicine.

Intervention	Main Objective	Examples in Veterinary Practice	Expected Outcomes
Clinical guidelines	Improve evidence-based prescribing [33,70]	ISCAID, WSAVA, BSAVA recommendations	Increased guideline adherence [39,51,67]
Diagnostic stewardship	Improve antimicrobial targeting [56,57,58] *	Culture, susceptibility testing, cytology, rapid diagnostics	Reduced empirical prescribing [59,60,67] *
Education and training	Improve knowledge and confidence [34,35]	Workshops, online modules, continuing education	Improved prescribing decisions [39,51,52]
Audit and feedback	Modify prescribing behavior [61,62,64] *	Peer comparison, benchmarking, individualized feedback	Reduced inappropriate prescribing [51,52,86]
Clinical decision-support systems	Support point-of-care decisions [50,66,87] *	Electronic prescribing tools, algorithms, AI-based systems	Reduced prescribing variability [65,66] *
Multifaceted programs	Address multiple barriers simultaneously [70,84]	Education + guidelines + audit + diagnostics	Sustained stewardship improvements [39,51]

* Evidence marked with an asterisk is derived from human healthcare literature. These studies are included where direct evidence from companion-animal practice is limited and are used to inform transferable behavioral, organizational, diagnostic, or antimicrobial stewardship concepts; they should not be interpreted as direct evidence for companion-animal practice.

**Table 3:** Evidence supporting antimicrobial stewardship effectiveness in companion animal practice.

Outcome Assessed	Current Evidence	Main Limitations	Future Research Needs
Antimicrobial prescribing frequency	Most studies report reductions after stewardship interventions [39,51,52,82]	Mainly observational studies	Multicenter controlled studies [97,98]
Prescribing appropriateness	Improvement reported after guideline-based interventions [39,51,67,82]	Lack of standardized assessment tools [97,98]	Validated quality indicators [80] *, [98]
Clinical outcomes	No consistent evidence of poorer outcomes following reduced prescribing [39,51,52,82]	Limited long-term follow-up [97,98]	Prospective longitudinal studies [97,98]
Antimicrobial resistance	Evidence remains limited [49,68,97]	Resistance changes require prolonged surveillance [68,97]	Genomic and One Health approaches [13,14,15,68,98]
Economic outcomes	Potential reduction in unnecessary costs [9,82]	Few veterinary cost-effectiveness studies [97,98]	Comprehensive health-economic analyses [9], [27,83,95] *

* Evidence marked with an asterisk is derived from human healthcare literature. These studies are included where direct evidence from companion-animal practice is limited and are used to inform transferable behavioral, organizational, diagnostic, or antimicrobial stewardship concepts; they should not be interpreted as direct evidence for companion-animal practice.

**Table 4:** Current knowledge gaps and future research priorities.

Knowledge Gap	Current Limitation	Recommended Research Direction
Standardization of outcomes	Different definitions of stewardship success [80] *, [97,98]	Development of core outcome sets [80] *, [97,98]
First-opinion practice evidence	Most studies from referral/university hospitals [39,51,52,98]	Pragmatic multicenter studies [97,98]
Long-term sustainability	Limited follow-up periods [39,51,52,82,97]	Longitudinal implementation studies [89,97,98]
AMR impact	Few studies link AMS with resistance reduction [49,68], [83,95] *	Integrated microbiological surveillance [13,19,20,24,68,98]
Digital stewardship	Limited veterinary validation [50], [99,100,101] *	AI and electronic health record studies [50], [99,100,101] *
Owner and clinician perspectives	Behavioral factors insufficiently explored [34,35,36,55], [38] *	Mixed-method implementation research [35,36,89,98]

* Evidence marked with an asterisk is derived from human healthcare literature. These studies are included where direct evidence from companion-animal practice is limited and are used to inform transferable behavioral, organizational, diagnostic, or antimicrobial stewardship concepts; they should not be interpreted as direct evidence for companion-animal practice.

## Data Availability

No new data were created or analyzed in this study. Data sharing is not applicable to this article.

## Abbreviations

The following abbreviations are used in this manuscript:

AMR	Antimicrobial resistance
WHO	World Health Organization
WOAH	World Organisation for Animal Health
FAO	Food and Agriculture Organization
MRSP	Methicillin-resistant *Staphylococcus pseudintermedius*
MRSA	Methicillin-resistant *Staphylococcus aureus*
ESBL	Extended-spectrum β-lactamase
AMS	Antimicrobial stewardship
ISCAID	International Society for Companion Animal Infectious Diseases
WSAVA	World Small Animal Veterinary Association
BSAVA	British Small Animal Veterinary Association
FECAVA	Federation of European Companion Animal Veterinary Associations
IPC	Infection prevention and control
MALDI-TOF	Matrix-assisted laser desorption/ionization time-of-flight
CDSS	Clinical decision-support systems
